# Reproductive and developmental safety assessment of Ashwagandha (*Withania somnifera*) root extract in Wistar rats

**DOI:** 10.3389/fphar.2025.1572025

**Published:** 2025-06-18

**Authors:** K. Kalaiselvan, V. Sweatha, V. Gayathri, P. Kalaivani, R. Siva, Shonam Tamrakar

**Affiliations:** Center for Toxicology and Developmental Research, Sri Ramachandra Institute of Higher Education and Research, Chennai, Tamil Nadu, India

**Keywords:** Ashwagandha root extract, developmental toxicity, reproductive toxicity, wistar rats, safety

## Abstract

**Introduction:**

Ashwagandha (*Withania somnifera* L. Dunal), a popular botanical drug in Ayurveda, has a wide range of therapeutic applications. However, safety data for effects on reproduction and embryonic development in animal models is scarce. This randomized controlled study investigated the effects on body weight, reproductive organ weight, and thyroid hormone levels in pups of Wistar rats (10M/13F per group) who received oral aqueous Ashwagandha Root extract (500, 1,000, or 2,000 mg/kg/day body weight) or carboxymethylcellulose (control) for 4 weeks.

**Methods:**

Animals were housed in polypropylene cages with males and females together during the pre-exposure and pre-mating periods. During the mating phase, one male and one female were kept together until confirmation of pregnancy. Pregnant females were housed individually during the post-mating period and lactating phase, while male rats were returned to their group cages. The procedure used was based on OECD Guideline 421 (OECD, 2016).

**Results:**

The No Observed Adverse Effect Level (NOAEL) in adult rats was 2,000 mg/kg. The mean body weights of pups and parental animals were similar (p > 0.05) in the treatment and control arms. Also, the weight of reproductive organs in parental male and female rats was similar (p > 0.05) in all groups.

**Discussion:**

The parameters such as T4 and TSH levels, body weight, and weight of reproductive organs in adult Wistar rats and pups, were within range even at the maximum dosage. No evidence of toxicity at various stages of development and reproduction with aqueous extract was observed when administered orally. Rats’ ability to reproduce and develop was unaffected by standardized ashwagandha root extract.

## 1 Introduction

Ashwagandha or *Withania somnifera*, Linn. Dunal remains one of the most highly sought botanical specimens due to its medicinal versatility in Ayurveda and Unani systems of medicine. (Bashir et al., 2023; Bhosale and Prajapati, 2022; Patel et al., 2016). This woody shrub belongs to the Solanaceae family (Solanaceae *Juss.*), which currently includes 84 genera and about 3,000 species. (Afewerky et al., 2021). This plant thrives in Mediterranean arid areas and tropical African regions, although it has also been reported to thrive in Asia, Australia, and Europe (Verma et al., 2021). Currently, Ashwagandha is a highly-sought plant-dried nutritional component. (Gómez Afonso et al., 2023). Traditionally, Ashwagandha is used to promote sleep and normalize bodily functions during stressful conditions (Kaushik et al., 2017).

Ashwagandha root extract (ARE) is also popularly known to exhibit aphrodisiac and testosterone-increasing properties (Abdelwahed et al., 2023). In some studies, ARE is classified as an adaptogen that acts on the axis between the hypothalamus gland and pituitary gland, the nervous system, and the endocrine system to promote bodily invigoration and reproductive rejuvenation (Prabu and Panchapakesan, 2015). The pharmacologic properties of Ashwagandha have been attributed to its bioactives such as alkaloids, phenolic metabolites, flavonoids, steroidal lactones, carbohydrates, and amino acids (Afewerky et al., 2021), although most of its therapeutic effects have largely been attributed to withanolides and sitoindoside (Girme et al., 2020). Withanolides are naturally occurring high-molecular weight lactones with an ergostane framework (Gupta et al., 2022; Wang et al., 2021). Sitoindosides, on the other hand, are withanolide glycosides, and include sitoindoside IX and sitoindoside X (Mikulska et al., 2023).

Data on toxicity provides future insights into the safety and desired dosage that is fit for human use (Gupta et al., 2022). A study conducted in India on a group of 80 fully healthy individuals confirmed the lack of toxicity of this raw material. The participants were each administered 300 mg of ARE orally, twice daily, for 8 weeks. Prabu et al. (2012) also noted that ARE did not exhibit toxicity to rats based on body weight parameters, organ weight changes, and blood biochemistry (Prabu et al., 2013). In the study of Patel et al. (2016), the orally administered ARE did not induce acute or sub-acute toxicity using morphologic, hematological, serum chemistry, and histopathological parameters in healthy adult Wistar rats (Patel et al., 2016). In a recent study, the No Observed Adverse Effect Level (NOAEL) of ARE was 2,000 mg/kg body weight/day in rats after repeated oral administration for 90-day (Kalaivani et al., 2023). Langade D. et al. (2023) reported that with 28-day repeated dose administration, Ashwagandha root extract does not show any major abnormalities in rats with doses up to 5 times of the recommended human dose. These findings substantiate the safety of ARE (Langade et al., 2023).

Despite several reports on the various pharmacologic effects and acute and sub-acute toxicity in animal models, there is a paucity of information regarding the toxicity of orally administered ARE to animal models during reproduction and development. Toxicity evaluation of the root extract during the reproductive and lactating stages of parental Wistar rats, and during the developmental period of their offspring offers valuable insights regarding the extract’s adverse effects on developing rat embryos during prenatal exposure and ensures the safety of the extract before human use (OECD, 2016). To the author’s knowledge, no study has provided additional documentation on the reproductive and developmental toxicity of ARE in rats. Hence, this study determined the effects of the daily oral administration of ARE on the body weights, organ weights, and thyroid hormone levels of adult parental rats and their pups.

## 2 Materials and methods

### 2.1 Test substance

**FIGURE 1:**
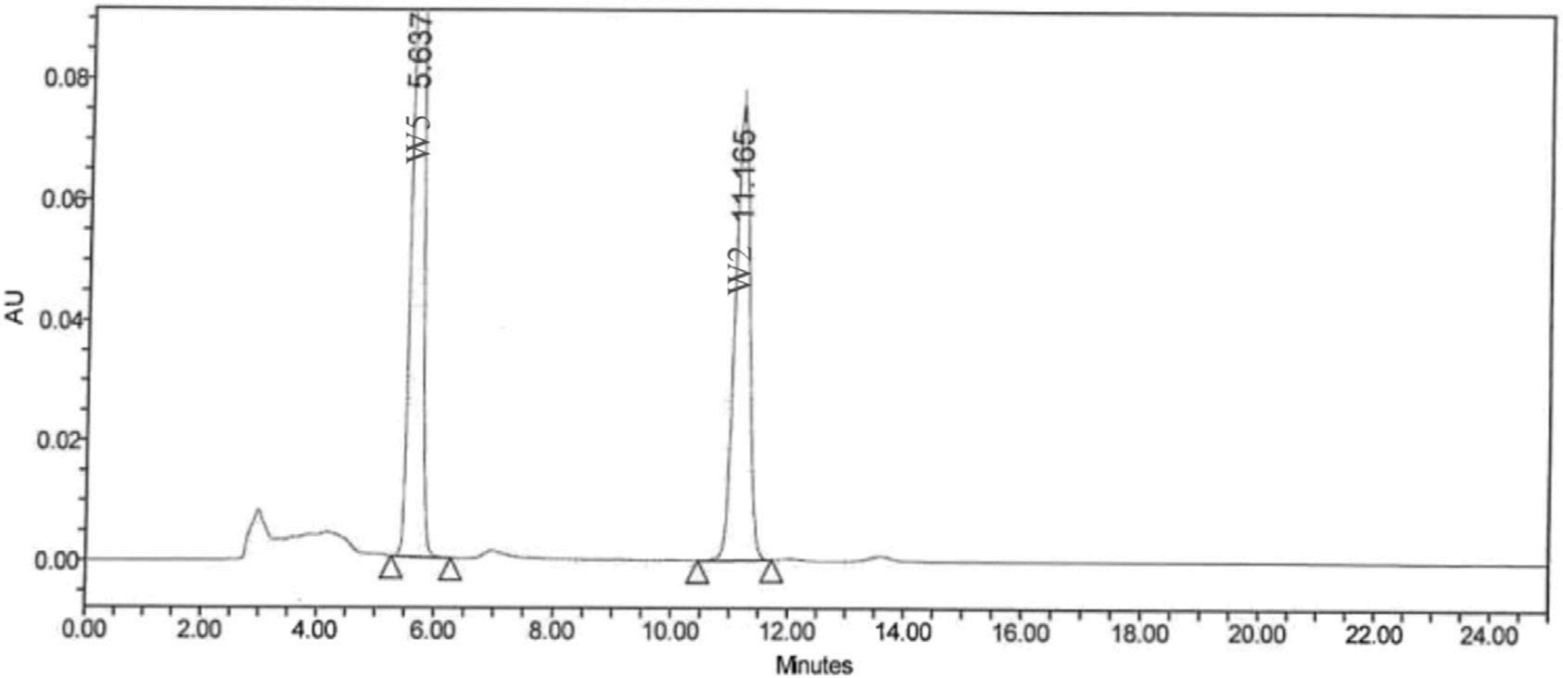
Chromatogram of HPLC analysis.

Ashwagandha root extract (KSM-66^®^) from Shri Kartikeya Pharma, Telangana, India (Batch KSM/22/340), follows modern approved Good Agricultural and Collection Practices (GACP), Current Good Manufacturing Practices (cGMP), Current Good Laboratory Practices (cGLP) which are used to maintain the quality of each batch of KSM-66 produced. KSM-66 is the commercially available highest concentration root-only extract of Ashwagandha which is produced through a green chemistry method that is devoid of any alcohol or chemical solvents. The process involves cleaning, high-temperature heating, and drying of the roots. After mixing with hot water and a 4-h extraction, the liquid undergoes 60-min heating in a reactor. The resulting decoction is dried in a tray dryer to achieve 2%–3% moisture content, forming a powder. Post-processing, samples undergo heavy metal, phytochemical, and microbiological analyses. The standardized extract contains >5% total withanolide content, confirmed by HPLC (Bashir et al., 2023). The analysis of withanolides was performed on a Waters HPLC system, using a C18 5 μm column of dimensions 250 × 4.6 mm, with a flow rate of 1 mL/min. The solvent system is based on methanol: water (65:35). At the end of the run, the column was flushed with 100% methanol for 30 min. The column temperature was 30°C, and the injection volume was 20 μL. Six withanolides were used as marker compounds in this study. 5 mg of a particular marker compound was accurately weighed and transferred into a 50 mL volumetric flask. 50 mL of HPLC grade methanol was poured into this volumetric flask, and the solution was sonicated for 15 min or until the compound dissolved completely. From this resultant solution, 1 mL, 2 mL, 3 mL, and 4 mL solutions were transferred into each of four different 10 mL volumetric flasks. The solution was filled up to the mark of each of the flasks with HPLC grade methanol to get a concentration of 10 ppm, 20 ppm, 30 ppm, and 40 ppm, respectively. 50 mg of powdered KSM-66 root extract was transferred to a 50 mL volumetric flask, and about 45 mL of methanol was added to it. It was sonicated for 30–45 min with gentle heat in an ultrasonic bath. The solution was finally made up to 50 mL. Before injection into the HPLC, this clear solution was filtered using a 0.22 μm syringe filter membrane. The product, which is slightly hygroscopic and yellowish-brown, is obtained through aqueous-based extraction. The drug to extract ratio is 12:1. The herbs are grown in regions with optimum rainfall (650 mm–750 mm), and proper soil condition (pH 7.5–8.0). Further, all steps of processing are done under proper approved guidelines, and the final product of each batch is subjected to Organoleptic testing, Moisture content analysis, microscopic analysis, microbiological analysis, Ash and pH testing, Aflatoxin testing, heavy metals and pesticide content analysis, and bioactive analysis. Figure 1 presents the chromatogram of HPLC analysis. The test product is distinguished under Extract type A as per Phytochemical Characterisation of Medicinal Plant extracts (ConPhyMP) guidelines.

### 2.2 Preparation of test solution

The root extract was homogenized in a mortar with a pestle and dissolved in 0.1% carboxyl methyl cellulose (CMC) to prepare a solution. The required volume of vehicle was added to make a fine, pasty liquid, and then it was transferred to a measuring cylinder to make up the final volume of formulation and transferred to a labelled beaker for dosing. Freshly prepared solutions were used to administer a low dose (Group 1:500 mg/kg bw), a medium dose (Group 2:1,000 mg/kg bw), and a high dose (Group 3:2,000 mg/kg bw) to the test animals. Solution homogeneity and uniformity were maintained through constant stirring after preparation until dosing.

### 2.3 Animal husbandry and maintenance

Vivo Bioscience, Bangalore, provided 92 healthy young adult female and male Wistar rats (*Rattus norvegicus*). After 5 days of acclimatization, suitability was confirmed. Rats were randomly assigned to low, medium, high-dose, and control groups. Each group included 10 fertile males and 13 nulliparous, non-pregnant females, selected based on stratified body weight. Rats were marked for identification, and cages were labeled. Doses did not exceed 10.0 mL/kg bw. The observation lasted 63 days, following OECD Guideline 421. Rats were housed in polypropylene cages with stainless-steel grids, maintained at temperatures of 19.01°C–22.95°C and relative humidity of 43.0%–69.2%. *Ad libitum* feeding with approved pellets and UV-irradiated water was provided. The Institutional Animal Ethics Committee approved the study (IEAC/62/SRIHER/708/2020), following care guidelines from the National Research Council.

### 2.4 Dose administration

Parental male and female Wistar rats in the low-dose group received 500 mg/kg bw of the extract, while rats in the medium-dose and high-dose groups received 1,000 mg and 2,000 mg per kg body weight, respectively. On the other hand, the rats in the control group received a 0.1% CMC solution. The extract was introduced via oral route using gavage once a day, for 28–63 days. Male rats were orally administered the extract daily for a period of 4 weeks, inclusive of the day before sacrifice. Female rats, on the other hand, were orally administered daily for 63 days during pre-mating and lactation.

### 2.5 Reproduction and development

During the 2 weeks of pre-mating and 2 weeks of mating, the estrous cycles of female rats were assessed. A minimum of 12 females per group, exhibiting the typical 4–5-day estrous cycle, were included. Each female in the treatment group was paired with a fertile male from the same treatment. Detection of sperm cells in the vaginal plug confirmed successful mating, recorded as day ‘0’ of pregnancy. Females showing no signs of copulation up to 26 days post-mating were euthanized.

### 2.6 Offspring parameters

In each group, the number of male and female pups was recorded during the postpartum period. To document stillbirths and live births, the pups were examined post-delivery. Other parameters, such as litter size, individual weight, and anogenital distance, were recorded during the first, fourth, and 13th postpartum days.

### 2.7 Evaluation of body weight

The body weights of male and female parental rats were recorded before orally administering the root extract and weekly thereafter during the pre-mating day and mating day. For female rats, the body weights were also measured weekly during pre-exposure (14 days), gestation (20 days), and lactation (13 days). The pup’s mean body weights were recorded weekly at 7-day intervals during postpartum period. The body weight changes of all the animals were calculated using the formula:

$$\%\;\text{Change}\;\text{in}\;b\;\text{wt}=\frac{\text{Final}\;b\;\text{wt}-\text{Initial}\;b\;\text{wt}}{\text{Initial}\;b\;\text{wt}}\;x\;100$$

### 2.8 Evaluation of toxicity

Mortality and morbidity were observed twice daily throughout the experimental period from the first day of acclimatization until the day of necropsy. Weekly body weight and body weight changes were recorded for all control and treatment groups. The pups were also observed for clinical signs such as hair growth, eye-opening, pinna unfolding, ear opening, the upper and lower eruption of the incisor, vaginal opening, and areolae. Necropsy was performed on deceased animals. As per the scheduled duration, animals were anesthetized for blood collection for hormonal analysis and sacrificed for gross examination. The gross pathological features and changes in organ weight were recorded for all test animals, and histopathological features of the ovaries, testes, and epididymis were noted.

### 2.9 Thyroid hormone analysis

Blood samples were collected from all male rats, dams, and pups 28 days post-dosing, day 63, and day 13, respectively. Through centrifugation, the serum was separated from whole blood at 3,500 rpm for 10 min at 28 ± 2°C and, then stored at −75 ± 5°C until analysis of serum thyroid hormones. The measurement of thyroid hormones such as thyroid stimulating hormone (TSH) and thyroxine (T4) was performed using an ELISA kit (T4 Kit - Krishgen Lot No. RT40423-1 and TSH Kit - Krishgen Lot No. RTSH0423-1- Expiry Date - 31.03.2024). The primary goal in diagnosing hypothyroidism is to confirm that there is an elevated TSH level (which indicates the thyroid is underactive) and that T4 levels are low, as this directly indicates thyroid dysfunction, thus T3 levels were not measured.

### 2.10 Necropsy and gross pathology

Adult rats were euthanized using xylazine sedation followed by CO_2_ asphyxiation, while pups were euthanized with isoflurane and cervical dislocation. All animals underwent gross necropsy, and pathology findings were documented. Male rats had their reproductive organs and associated structures, including the testes, levator ani, bulbocavernosus muscle complex, Cowper’s gland, glans penis, and epididymides, trimmed and weighed immediately. These organs were then preserved in modified Davidson’s fluid for subsequent analysis. Female rats’ thyroid glands and sex organs, such as the ovaries, uterus, and cervix, were collected, fixed in 10% neutral buffer formalin, and weighed after gross pathological examination. Thyroid glands were weighed post-fixation. The individual terminal body weight of all surviving animals was recorded and used to calculate organ weight relative to the terminal body weight using the formula:

$$\text{Organ}\;\text{Weight}\left(\%\right)\;\text{relative}\;\text{to}\;\text{terminal}\;b\;\text{wt}=\frac{\text{Weight}\;\text{of}\;\text{organ}\;\left(g\right)}{\text{Terminal}\;b\;\text{wt}\;\left(g\right)\;\text{of}\;\text{the}\;\text{animal}}\;x\;100$$

### 2.11 Histopathology

A detailed histopathological examination was performed on the reproductive and other accessory organs to determine the stage of spermatogenesis. The reproductive organs of the adult Wistar rats were trimmed, processed, and then embedded in paraffin blocks. After which, the tissues were sectioned into 4–6 microns. Staining was performed using the hematoxylin and eosin methods to evaluate histopathology of the tissues. The rats’ thyroid glands were not examined, as there is no endocrine disruptor change in thyroid hormones.

### 2.12 Statistical analysis

Statistical Package for Social Sciences (SPSS) version 29 for Windows (Armonk, NY: IBM Corp.) was used to perform analysis of body weight, reproductive and accessory organ weights, anogenital distance, and serum thyroid hormone levels. A one-way analysis of variance (ANOVA) with *post hoc* Fisher LSD was utilized to determine the significant mean differences in the control group and the three treatment groups. Prior to conducting the ANOVA, Shapiro-Wilk’s test for normality and Levene’s test were conducted. A *p*-value <0.05 was considered statistically significant.

## 3 Results

### 3.1 Effects of ashwagandha root extract on reproduction and development

Table 1 shows a summary of the effects of the orally administered ARE on the test animals. There was no mortality observed among male rats during treatment. Although treatment-related mortality was not observed in adult female rats, there were a few cases of mortality control group = 2, Group 1 = 1, Group 2 = 1, Group 3 = 2 after the delivery of pups. Furthermore, no mortality was observed in the newly born male and female pups. However, mortality was observed during lactation in all groups from day 0 to day 4, although the deaths were not attributed to treatment-related interventions.

**TABLE 1:** Summary of effects of Ashwagandha root extract on male and female rats.

Parameter	Reproductive and developmental values			
	Control	Group 1	Group 2	Group 3
	0.1% CMC	500 mg/kg bw	1,000 mg/kg bw	2,000 mg/kg bw
Number of male rats (N)	10	10	10	10
Number of female rats (N)	13	13	13	13
Female rats showing evidence of copulation (N)	13	13	13	12
Female achieving pregnancy (N)	13	13	13	12
Pregnancy Index (%)	100%	100%	100%	92%
Pregnant rats during days 1–5 post-conception (N)	13	13	13	12
Pregnant rats during days 6–21 post-conception (N)	12	13	12	10
Pregnant rats equal during day 21 or greater (N)	12	12	9	10
Pregnant rats at 22 days (N)	1	1	3	0
Number of dams with live pups at day 0 (N)	8	8	7	8
Number of dams with live pups at day 4 (N)	5	6	6	6
Number of implants (N)	121	115	117	99
Mean implants/dam	9.30	8.84	9.00	8.25
Live pups at birth (N)	72	68	57	66
Mean live pups per dam at birth	91.38	98.75	98.21	94.32
Mean live pups at day 4	45	50	52	46
Mean live pups per dam at day 4	97.98	100	98.33	95.83
Mean sex ratio* at birth	164.6	110.9	138.8	174.0
Mean sex ratio* at day 4	198.3	125.0	145.3	175.0
Mean weight of litter at birth (g)	53.66	49.48	50.65	47.07
Mean weight of litter (day 4) (g)	73.31	77.82	80.27	66.75
Mean weight of pups at birth (g)	5.98	5.78	6.18	5.65
Mean weight of pups (day 4) (g)	8.33	9.46	9.51	8.73
Mean weight of pups at day 13 (g)	19.35	20.48	23.43	19.79
Stillbirth and runts (N)	7	1	1	5
Anogenital distance of pups at day 0 (M ± SD)	3.24 ± 1.24	3.33 ± 1.28	3.12 ± 1.37	3.78 ± 1.47
Anogenital distance of pups at day 4 (M ± SD)	4.54 ± 1.54	1.40 ± 4.24	4.24 ± 1.65	3.91 ± 1.45

Abbreviations: bw, body weight; CMC, carboxymethylcellulose.

^a^ Sex ratio = (no. of live male pups/no. of live female pups) x 100.

### 3.2 Effects of ashwagandha root extract on body weight of male and female adult rats

The summary of male and female rats’ body weights is summarized in Table 2. Overall, male rats’ mean body weight increased in all groups. The test animals in Group 2 exhibited a decrease in body weight during days 0–7 (M = −4.80, SD = 30.30), and days 7–14 (M = −1.40, SD = 9.98). Test animals in group 3 also exhibited a slight decrease in their mean body weight from day 7 to day 14 (M = −0.40, SD = 28.57). Throughout the duration of the experiment, the mean body weights of male rats in all groups did not exhibit statistically significant differences (*p* > 0.05). Similarly, female rats’ mean body weights during pre-exposure, pre-mating, gestation, and lactation stages were statistically comparable (*p* > 0.05).

**TABLE 2:** Average weight of male and female rats by dosage group during experiment.

Day	Control (C)	Group 1 (G1)	Group 2 (G2)	Group 3 (G3)	F	*p*-value
	0.1% CMC	500 mg/kg bw	1,000 mg/kg bw	2,000 mg/kg bw		
Male rats						
N	10	10	10	10		
Day 0	305.30 ± 49.81	310.60 ± 35.11	313.80 ± 38.62	307.00 ± 34.29	0.090	0.965
Day 7	312.40 ± 51.36	301.00 ± 42.43	323.80 ± 35.73	315.10 ± 33.88	0.516	0.674
Day 14	329.10 ± 66.00	304.40 ± 41.36	323.40 ± 28.35	317.80 ± 34.72	0.554	0.649
Day 21	323.70 ± 51.05	301.40 ± 45.77	326.90 ± 29.70	311.10 ± 36.91	0.797	0.504
Day 28	333.70 ± 51.10	320.30 ± 39.80	333.80 ± 28.54	323.10 ± 53.00	0.254	0.858
Female rats (pre-exposure)						
N	13	13	13	13		
Day 0	208.85 ± 14.21	209.15 ± 14.09	210.23 ± 14.56	206.00 ± 11.78	0.213	0.887
Day 7	214.08 ± 11.93	213.92 ± 17.89	214.54 ± 14.12	214.58 ± 12.06	0.007	0.999
Day 14	219.77 ± 15.69	220.54 ± 19.20	220.38 ± 15.86	218.00 ± 11.97	0.065	0.978
Female rats (pre-mating)						
N	13	13	13	13		
Day 0	223.92 ± 15.38	225.54 ± 21.72	226.00 ± 16.69	222.58 ± 15.00	0.100	0.959
Day 7	228.31 ± 16.53	223.00 ± 19.75	231.46 ± 17.91	225.75 ± 16.98	0.530	0.664
Day 14	226.38 ± 17.21	225.85 ± 21.23	228.77 ± 18.33	227.92 ± 15.76	0.070	0.975
Female rats (gestation)						
N	13	13	12	11		
Day 0	228.69 ± 17.43	227.92 ± 25.02	213.85 ± 67.40	230.36 ± 16.80	0.497	0.686
Day 7	238.46 ± 27.59	242.54 ± 29.63	246.31 ± 22.90	252.83 ± 27.72	0.634	0.597
Day 14	269.83 ± 30.89	262.85 ± 35.36	269.38 ± 29.41	271.91 ± 17.70	0.22	0.882
Day 20	304.08 ± 40.52	296.08 ± 47.44	308.50 ± 41.03	309.50 ± 34.37	0.263	0.852
Female rats (lactation)						
N	11	12	11	8		
Day 0	220.64 ± 18.96	232.00 ± 29.21	236.00 ± 32.63	232.75 ± 9.02	0.768	0.519
Day 4	227.45 ± 28.54	243.64 ± 31.19	240.18 ± 32.89	244.75 ± 10.44	0.827	0.487
Day 13	252.70 ± 19.02	251.64 ± 27.16	255.64 ± 28.02	259.63 ± 11.84	0.214	0.886

Values are presented as mean ± standard deviation (M ± SD).

Number of test animals (N) during lactation and gestation.

Control group (gestation: day 7 = 13, day 14 = 12, day 20 = 12; lactation: day 0 = 11, day 4 = 11, day 13 = 10).

Low-dose group (gestation: day 7 = 13, day 14 = 13; day 20 = 13; lactation: day 0 = 12, day 4 = 11, day 13 = 10).

Medium-dose group (gestation: day 7 = 13, day 14 = 13; day 20 = 12; lactation: day 0 = 11, day 4 = 11, day 13 = 11).

High-dose group (gestation: day 7 = 12, day 14 = 11; day 20 = 10; lactation: day 0 = 8, day 4 = 8, day 13 = 8).

### 3.3 Effects of ashwagandha root extract on post-natal mean weight of pups

Table 3 shows the average weight of pups in all dosage groups. Overall, the pups’ weekly mean weights increased up to day 13. On day 0, the mean body weight of pups in the medium-dose group was higher compared to other groups (*p* < 0.001). However, the weight of pups in the other groups was similar (*p* > 0.05). At day 4, the mean weight of pups in the control group was lower (*p* > 0.05) compared to other dosage groups, while the pups in the three remaining groups did not exhibit a statistically significant difference in the mean weight (*p* > 0.05).

**TABLE 3:** Average weight of pups by dosage group.

Post natal day	Control	Group 1	Group 2	Group 3	F	*p*
	0.1% CMC	500 mg/kg BW	1,000 mg/kg BW	2,000 mg/kg BW		
N	72	68	57	66		
Day 0	5.96 ± 0.73	5.82 ± 0.73	6.22 ± 0.67	5.71 ± 0.55	6.489	<0.001
N	45	50	52	46		
Day 4	8.15 ± 1.19	9.41 ± 1.53	9.26 ± 1.19	8.71 ± 1.42	8.742	<0.001
N	38	44	43	41		
Day 13	22.16 ± 16.11	21.17 ± 4.22	23.11 ± 2.97	21.50 ± 2.33	0.464	0.708

Abbreviations: bw, body weight; CMC, carboxymethylcellulose.

### 3.4 Effect of ashwagandha root extract on the terminal and relative organ weight

Table 4 shows the body and organ weights of parental test animals on the last day of treatment. The highest mean terminal body weight of male rats was noted in the medium-dose group, followed by the control group, the high-dose group, and then the low-dose group, although the differences are minor and statistically insignificant (*p* > 0.05). However, the mean weight of the levator ani and bulbocavernosus muscle complex was statistically significantly higher in the medium-high dose group compared to other treatment groups and the control group (*p* < 0.001). Among female rats, the highest mean terminal body weight was noted in the high-dose group, followed by the medium-dose group, control group, and low-dose group, although the means were not statistically significantly different (*p* > 0.05).

**TABLE 4:** Terminal body weight and organ weights of male and female rats.

Parameters	Control	Group 1	Group 2	Group 3	F	*p*
	0.1% CMC	500 mg/kg bw	1,000 mg/kg bw	2,000 mg/kg bw		
Male rats (N)	10	10	10	10		
Terminal body weight (g)	333.70 ± 1.10	320.30 ± 9.80	337.30 ± 8.12	323.10 ± 3.00	0.344	0.794
Thyroid + parathyroid gland (mg)	18.80 ± 4.16	18.80 ± 2.86	20.40 ± 4.40	21.40 ± 2.55	1.275	0.297
Testes (g)	3.37 ± 0.33	3.32 ± 0.38	3.24 ± 0.40	3.15 ± 0.50	0.608	0.614
Male sex gland (g)	2.47 ± 0.46	2.43 ± 0.35	2.50 ± 0.47	2.51 ± 0.28	0.081	0.97
Epididymis (g)	1.41 ± 0.12	1.52 ± 0.23	1.53 ± 0.25	1.36 ± 0.15	1.716	0.181
LA + BC (g)	1.15 ± 0.22	1.07 ± 0.09	1.46 ± 0.14	1.35 ± 0.22	10.564	<0.001
Glans penis (g)	0.13 ± 0.04	0.13 ± 0.02	0.12 ± 0.03	0.11 ± 0.03	0.32	0.811
Cowper’s gland (g)	0.10 ± 0.04	0.10 ± 0.02	0.26 ± 0.43	0.11 ± 0.03	1.275	0.297
Female rats (N)	46	46	46	46		
Terminal body weight (g)	250.45 ± 9.41	245.50 ± 8.93	253.42 ± 7.90	258.45 ± 5.39	0.508	0.679
Thyroid + parathyroid gland (mg)	15.27 ± 1.90	15.08 ± 2.87	17.17 ± 3.01	17.82 ± 3.49	2.560	0.068
Ovaries (g)	0.13 ± 0.03	0.25 ± 0.39	0.13 ± 0.04	0.14 ± 0.03	1.035	0.387
Uterus + cervix (g)	0.45 ± 0.17	0.55 ± 0.19	0.48 ± 0.17	0.49 ± 0.36	0.344	0.794

Data form organ weights were presented using mean ± standard deviation.

Abbreviations: LC + BC, levator ani + bulbocavernosus muscle complex; mg/kg bw, milligram per kilogram body weight.

Table 5 summarizes the relative organ weight of parental rats. All relative mean organ weights of adult test animals in the treatment groups were comparable to the control group (p > 0.05). Furthermore, no treatment-related histopathological findings on the ovaries, testes, and epididymis were noted even if the dose was 2,000 mg/kg bw, which was the maximum dosage in this study.

**TABLE 5:** Relative organ weight (grams) of male rats after administration of Ashwagandha root extract.

Parameters	Control	Group 1	Group 2	Group 3	F	*p*
	0.1% CMC	500 mg/kg bw	1,000 mg/kg bw	2,000 mg/kg bw		
Male rats (N)	10	10	10	10		
Thyroid + parathyroid gland	5.60^−2^ ± 1.26^−2^	6.00^−2^ ± 0.47^−2^	6.00^−2^ ± 1.41^−2^	6.70^−2^ ± 1.25^−2^	1.553	0.218
Testes	1.03 ± 0.19	1.05 ± 0.15	0.97 ± 0.14	1.00 ± 0.21	0.445	0.722
Male sex gland	0.74 ± 0.10	0.76 ± 0.08	0.75 ± 0.17	0.76 ± 0.13	0.049	0.986
Epididymides	0.43 ± 0.10	0.48 ± 0.09	0.46 ± 0.10	0.43 ± 0.09	0.554	0.649
LA + BC	0.35 ± 0.05	0.34 ± 0.04	0.44 ± 0.06	0.49 ± 0.35	1.758	0.173
Glans penis	0.04 ± 0.02	0.04 ± 0.01	0.04 ± 0.01	0.04 ± 0.02	0.233	0.873
Cowper’s gland	0.03 ± 0.01	0.03 ± 0.01	0.08 ± 0.15	0.04 ± 0.01	1.178	0.332
Female rats (N)	46	46	46	46		
Thyroid + parathyroid gland	6.10^−2^ ± 0.84^−2^	6.23^−2^ ± 1.45^−2^	6.80^−2^ ± 1.05^−2^	6.74^−2^ ± 1.16^−2^	1.078	0.369
Ovaries	50.66^−3^ ± 12.96^−3^	54.85^−3^ ± 12.82^−3^	50.76^−3^ ± 16.94^−3^	56.26^−3^ ± 14.56^−3^	0.444	0.723
Uterus + cervix	181.47^−3^ ± 67.25^−3^	227.77^−3^ ± 88.84^−3^	192.09^−3^ ± 68.89^−3^	220.35^−3^ ± 61.55^−3^	0.531	0.663

Data for organ weights (gm) were presented as mean ± standard deviation.

Abbreviations: LC + BC, levator ani + bulbocavernosus muscle complex; mg/kg bw = milligram per kilogram body weight.

### 3.5 Effect of ashwagandha root extract on thyroid hormones

**FIGURE 2:**
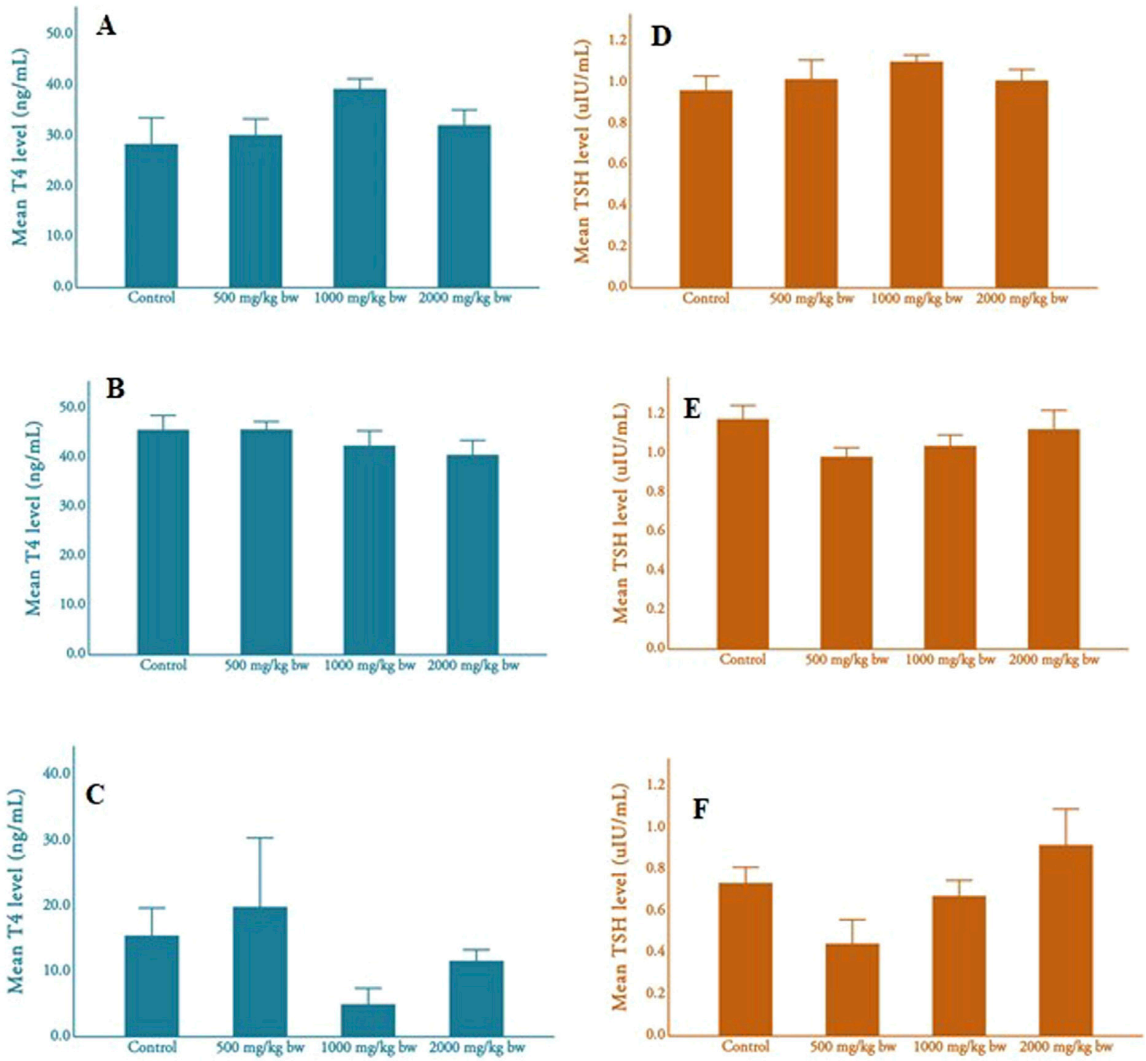
Mean T4 and TSH levels of adult rats and pups **(A)**, mean T4 level of male rats; **(B)**, mean T4 level of female rats; **(C)**, mean T4 level of pups **(D)**, mean TSH level of male rats; **(E)**, mean TSH level of female rats; **(F)**, mean TSH level of pups.

Figure 2 shows the thyroid hormone levels (e.g., thyroxine and thyroid stimulating hormone) among parental male rats, parental female rats, and pups. Thyroid hormone (T4) levels varied among parental male and female rats and their pups across different dosage groups. Male rats showed the highest T4 levels in the medium-dose group (M = 39.21 ± 6.64 ng/mL, N = 10), while females exhibited the levels in the low-dose group (M = 45.70 ± 5.58 ng/mL, N = 12). Pups displayed varied T4 levels, with the lowest in the medium and high-dose groups and the highest in the low-dose group (M = 15.47 ± 9.43 ng/mL, N = 5). No significant differences were found among adult male rats, adult female rats, and pups, and there was no clear dose-dependent pattern in T4 levels.

Thyroid stimulating hormone (TSH) levels also varied across dosage groups but showed minimal variation in parental male rats. Parental females displayed the highest TSH levels in the control group. Pups exhibited higher TSH levels in the medium-dose group compared to other groups. No significant differences were observed in TSH levels among parental males, parental females, and pups across different dosages, and there was no apparent dose-dependent pattern.

## 4 Discussions

The diverse applications of Ashwagandha root extract (ARE) have been highlighted in several studies, highlighting its wide range therapeutic applications. ARE has been reported to promote sleep induction and improve sleep management (Cheah et al., 2021; Choudhary et al., 2017) exhibit anticancer properties (Vaishnavi et al., 2012), regulate reproductive hormone levels (Ahmad et al., 2010), increase sperm count in animal models (Ambiye et al., 2013), improve mental alertness (Speers et al., 2021), promote neuroprotective effects (Elhadidy et al., 2018), protect cells from oxidative neurodegeneration (Wongtrakul et al., 2021), and improve cognitive functions. (Cheah et al., 2021). Administration of ARE increases serum testosterone and luteinizing hormone and reduces the levels of follicle-stimulating hormone and prolactin in male human participants. (Vaishnavi et al., 2012). To establish a baseline data set, this study investigated the effect of various doses (500 mg/kg bw to 2,000 mg/kg bw) of ARE via oral administration to male and female adult rats from pre-mating to postpartum periods and documented their potential impacts on toxicological parameters such as body weight, organ weight, and thyroid hormone levels. Furthermore, pregnant female rats in each group were continuously dosed with the root extract to determine the effects of oral administration of ARE on rat pups during the lactation phase.

Earlier efforts to investigate the toxicity of the ashwagandha extracts to rats have been done, (Sharada et al., 1993), although there was a need to further establish the effects of the continuous oral administration on the male and female offspring rats. This study evaluated various indicators of toxicity. Signs such as reduced body weight, lower organ weight, decreased food intake, and the presence of visible and microscopic tissue abnormalities suggest the potential toxicity of plant extracts. (Deng et al., 2023). ARE did not show any effect on thyroid gland in developing zebra fish embryos and has been reported to be safe with respect to its effects on thyroid gland. (Naseem et al., 2023). Plant metabolites, such as phenolics, flavonoids, and alkaloids may affect the thyroid peroxidase or type-1 deiodinase or prevent the mineralization of iodine in the thyroid cells. (Aleebrahim-Dehkordy et al., 2018). Any disruption of thyroid hormone levels such as thyroxine (T4) and thyroid stimulating hormone (TSH) may indicate the effect on thyroid functions. (Di Dalmazi and Giuliani, 2021). Mortality observed in female rats and pups during the study appeared to occur randomly, with no correlation to the administration of the root extract. Daily administration of the extract, even at the highest dose, did not significantly affect body weight, reproductive organ weight, or serum T4 and TSH levels in adult Wistar rats and pups. Furthermore, no histopathological evidence of toxicity was identified, suggesting that oral administration of the extract across various reproductive and developmental stages does not induce toxicity.

The prenatal toxicity of ARE among rats was described by Prabu and Panchapakesan (2014). (Prabu and Panchapakesan, 2015). Based on their study, there was no evidence of gross pathological malformations, skeletal and soft tissue malformation, and external malformations. The study found no histopathological signs of treatment-related toxicity in rat pups, suggesting that orally administered ARE is not toxic to developing embryos during pregnancy. These results imply potential non-toxicity of the root extract during pregnancy, although further clinical trials involving gene studies may be needed for confirmation. Regarding reproductive toxicity parameters, insignificant differences were observed among male rats in testes, male sex gland, epididymides, glans penis, and Cowper’s gland weights across control and treatment groups, even at high doses. Minor variations were noted in levator ani and bulbocavernosus muscle complex weights in male rats, but relative weights remained consistent. Similarly, female rats exhibited comparable relative reproductive organ weights across all dose levels.

The 0.1% CMC, the solution administered to the control group, and the solvent used for preparing the three doses of ARE does not cause toxicity to rats. Hence, the similarity of results in the control group and the different treatment groups provides an estimate on the relative non-toxicity of the root extract in the test animals. The results indicate the standardized ARE does not exhibit reproductive and development toxicity to Wistar rats.

The limitation of the study includes lack of positive control, and missing T3 (triiodothyronine) estimation. Additionally, the genotoxicity study was not done which might have served as an impactful factor in supporting the reproductive and developmental non-toxicity of ARE.

## 5 Conclusion

The findings of this investigation demonstrate the remarkable safety profile of orally administered Ashwagandha root extract, with a No Observed Adverse Effect Level (NOAEL) of 2,000 mg/kg bw. Importantly, the extract exhibited no toxicity to developing fetuses and pups when administered to adult male and female rats during reproduction. These results underscore the promising safety and potential therapeutic utility of Ashwagandha root extract in reproductive contexts.

## Data Availability

The raw data supporting the conclusions of this article will be made available by the authors, without undue reservation.
